# Extended indications for varicocelectomy

**DOI:** 10.12688/f1000research.19579.1

**Published:** 2019-09-03

**Authors:** G. Luke Machen, Jay I. Sandlow

**Affiliations:** 1 Department of Urology, Medical College of Wisconsin, 8701 Watertown Plank Road, Milwaukee, WI, 53226, USA

**Keywords:** varicocele, varicocelectomy, non-obstructive azoospermia, DNA fragmentation, hypogonadism

## Abstract

The relationship between varicoceles and subfertility is well-established, but recent evidence suggests that varicoceles may cause global testicular dysfunction. This has led to exploration into expanding the indications for varicocelectomy. This review examines the literature regarding varix ligation as a treatment for non-obstructive azoospermia, elevated DNA fragmentation, and hypogonadism.

## Introduction

Varicoceles remain the most common correctable cause of male infertility and have been identified in about 35 to 40% of men with primary infertility and up to 80% with secondary infertility ^1– 3^. The first study describing a potential improvement to fertility was published in 1885, when Barwell described improved testicular size after ligating dilated scrotal veins with wire loops ^4^. In the mid-20th century, several studies by Tulloch demonstrated improvements in semen parameters with varix ligation, essentially providing the foundation for modern thinking regarding varicoceles ^5, 6^. Now, it is established that repair of clinical, palpable varicoceles may provide significant improvements in semen parameters in men with subfertility ^7^. Concordantly, the American Society for Reproductive Medicine published guidelines recommending varix ligation in the presence of a palpable varicocele and semen parameter derangements ^8^. However, not all men with varicoceles have subfertility, and it remains somewhat of a challenge to identify who may derive the most benefit from operative intervention for varicoceles.

The mechanism through which varicoceles may cause testicular dysfunction is somewhat unclear. The most common hypothesis is testicular hyperthermia via disruption of the countercurrent heat-exchange system of the pampiniform plexus. Multiple studies have demonstrated that men with varicoceles have higher intra-scrotal temperatures ^9, 10^ and that these temperatures are lower after ligation. Other hypotheses include reflux of renal metabolites or hormonal disruptions or both ^11– 13^. Furthermore, men with varicoceles may have altered DNA and impaired sperm maturation via increased reactive oxygen species and decreased antioxidant capacity ^14, 15^.

Regardless of the mechanism, evidence suggests that varicoceles may cause global testicular dysfunction, including dysfunction of both Sertoli and Leydig cells ^16– 18^. Given this information, the utility of repairing varicoceles for alternative indications has been investigated. Here, the current support for these indications, including non-obstructive azoospermia (NOA), elevated DNA fragmentation, and hypogonadism, will be summarized.

## Discussion

### Non-obstructive azoospermia

Among men with NOA, varicoceles may be found approximately 4.3 to 13.3% of the time ^19^. Although it is unclear whether the presence of a varicocele may be incidental or causative, multiple studies have been performed to assess whether varix ligation leads to either return of sperm to the ejaculate or improved sperm retrieval rates in men with NOA.

A 2016 meta-analysis by Esteves *et al*. compiled 18 studies—seven prospective and 11 retrospective—of 468 patients with NOA and varicoceles ^20^. The authors found that 43.9% (range of 20.8 to 55.0%) of men with NOA had sperm present in their ejaculate following varix ligation ^20^. The highest rates of ejaculated sperm were identified in men with hypospermatogenesis (56.2%). Additionally, three out of 18 studies (n = 400) contained a control arm and were used to analyze fecundity outcomes. The authors found that sperm retrieval rates were increased (odds ratio [OR] 2.65, 95% confidence interval [CI] 1.69 to 4.14) after varicocelectomy and that increases in pregnancy rates and live birth rates approached statistical significance (OR 2.07, 95% CI 0.92 to 4.65 and OR 2.19, 95% CI 0.99 to 4.83, respectively) ^20^.

A 2016 meta-analysis by Kirby *et al*. evaluated the effect of varicocelectomy in azoospermic men prior to assisted reproduction ^21^. Although only two studies met criteria for inclusion, the authors found increased sperm retrieval rates (OR 2.51, *P* = 0.0001) and pregnancy rates (OR 2.34, 95% CI 1.02 to 5.34) among men with azoospermia after varix ligation. There was a trend toward improved live birth rates but this did not reach statistical significance (OR 2.21, 95% CI 0.99 to 4.90) ^21^.

Although more studies are needed, it does seem that varicocele ligation may be associated with increased rates of sperm in the ejaculate and improved fecundity outcomes in men with NOA. However, some caution is urged in interpreting these results. In fact, some reports have found that up to 35% of men with NOA may transiently have sperm in their semen analysis without any sort of treatment ^22^. Additionally, around 25% of men who regain sperm in their ejaculate following varicocelectomy regress to azoospermia on subsequent semen analyses ^23– 25^. Furthermore, Schlegel and Kaufmann published a study in which, although 22% of men with NOA gained sperm in their ejaculate after varix ligation, only 9.6% of the patients had sufficient motile sperm so as to avoid testicular sperm extraction ^26^. Thus, progression to assisted reproduction may be unnecessarily delayed in these couples where timing is frequently critical, particularly in couples with advanced female age.

However, the potential benefit of the return of ejaculated sperm should not be overlooked. Varicocelectomies in azoospermic men may obviate the need for an invasive procedure to harvest sperm and potentially lead to spontaneous pregnancy, as indicated by the 13.6% spontaneous pregnancy rate in the aforementioned review by Esteves *et al*. ^20^.

### DNA fragmentation

Despite having normal semen parameters, many men with varicoceles struggle to conceive. This led to the development of complementary testing to better determine who might benefit from surgical repair, including DNA fragmentation. Sperm DNA fragmentation has been shown to be associated with decreased fertility through inhibition of fertilization, embryo development, and implantation and lead to increased rates of miscarriage ^27– 29^.

Varicoceles are a well-established cause of sperm DNA damage ^30^. In fact, in a 2015 study of 593 men, varicoceles were associated with DNA fragmentation rates of 35.7% (standard deviation [SD] 18.3%), second only to men with leukocytospermia (41.7%, SD 17.6%); in the fertile controls, DNA fragmentation rates were 11.3% (SD 5.5%) ^31^. Although the mechanism through which varicoceles lead to DNA damage is not entirely clear, it is believed to be mediated through elevations in the number of reactive oxygen species and decreases in antioxidant capacity ^32– 34^. The resultant oxidative stress may lead to membrane lipid peroxidation, induction of apoptosis, and direct DNA damage ^35^.

Evidence suggests that DNA fragmentation may be reduced through repair of varicoceles. In 2011, Zini and Dohle published a review of 12 studies involving 511 patients; in all 12 studies, varix ligation was associated with decreases in sperm DNA damage ^30^. A meta-analysis published the following year by Wang *et al.* ^36^ found that DNA fragmentation improved by an average of 3.37% (95% CI 2.65 to 4.09%) following varicocelectomy. These results are bolstered by several recent randomized controlled trials. The first, published by Sun *et al*., reported reductions in DNA fragmentation index at 1 year post-operatively from 21.6 to 11.8% and from 23.0 to 12.1% for men undergoing unilateral and bilateral varicocelectomy, respectively ^37^. The same year, Zaazaa *et al*. reported improvements from 34.6 to 28.3% after subinguinal varix ligation ^38^.

Of note, although more studies are needed to completely characterize the effect of varix grade on DNA fragmentation, evidence suggests that higher-grade varicoceles may be associated with increased DNA damage ^39, 40^. Correspondingly, although DNA fragmentation has been shown to improve with all varicocele grades, larger decreases have been shown after repair of grade 3 varices ^38^. It is important to point out that although evidence is somewhat limited describing the effect of DNA fragmentation improvements after varix ligation on pregnancy rates, current data do suggest that DNA damage is decreased among couples who achieve pregnancy after varicocelectomy ^41, 42^. Regardless, at this time, it may be reasonable to consider DNA fragmentation testing in the setting of a grade 2 or 3 varicocele with normal semen parameters or a grade 1 varicocele with abnormal semen parameters ^43^.

### Hypogonadism

As mentioned previously, there is evidence that varicoceles may lead to impairment of Leydig cell function in addition to that of Sertoli cells. The first report describing improved testosterone levels with varix ligation dates back to 1975, when Comhaire and Vermeulen published a small series in which a hypogondal cohort had normalization of their testosterone levels after varicocelectomy ^44^. Several years later, Rodriguez-Rigau *et al*. found that men with varicoceles had decreased numbers of Leydig cells and there seemed to be a direct correlation between the degree of impairment of Leydig cells and spermatogenesis ^45^. Since that time, several other studies have demonstrated negative effects on Leydig cell function among men with varicoceles ^3, 16, 46^. More recently, Tanrikut *et al*. reported that men with varicoceles had testosterone levels that were significantly lower than those of controls (412.2 versus 462.2 ng/dL) ^47^.

These reports led to increased interest regarding the possible benefit of varix repair on hypogonadism, and in the late 20th century, several notable studies were published. Perhaps the first two of note were published by Su *et al*. (1995) ^48, 49^ and Cayan *et al*. (1999) ^48, 49^. These authors found that testosterone improved by 90 and 274 ng/dL, respectively, after varicocelectomy. Since the publishing of these studies, a growing body of evidence has suggested that varicocelectomy may improve testosterone production. A meta-analysis performed in 2012 found a mean improvement in testosterone levels of 97.5 ng/dL following varix ligation in 814 men ^50^. One criticism of that analysis was the marked heterogeneity of the included studies. To address this, Chen *et al*. performed a meta-analysis with a more stringent set of inclusion criteria ^51^. Ultimately, they used eight studies including 712 patients, and the overall improvement of testosterone among subfertile men undergoing varix ligation was 34.3 ng/dL (95% CI 22.57 to 46.04). However, the mean improvements were 123 ng/dL (95% CI 114.61 to 131.35) in hypogonadal (T <300 ng/dL) men and 12.73 ng/dL (−25.81 to 51.28) among eugonadal men who underwent varicocelectomy. These findings are in agreement with literature suggesting that men with low or low-normal testosterone may derive the greatest increases in testosterone from varicocelectomy ^52^.

Further studies have shown that varicocelectomy may lead to increases in testosterone. However, most studies were performed on men with subfertility and this may be a source of selection bias. Investigations of varix ligation purely as a treatment for hypogonadism are somewhat more rare. Two prospective studies found modest testosterone improvements in this patient population. Specifically, the baseline testosterone levels were 331 and 347, and improvements of 26 and 45 ng/dL, respectively, were identified ^53, 54^.

Given that these studies at times show modest testosterone improvements, it can be difficult to assess the clinical impact, although multiple studies have attempted to address this question. One report in 2011 described an improvement in International Index of Erectile Function survey scores among hypogonadal men who underwent varix ligation ^55^. A similar study in 2017 found statistically significant improvements in the Male Sexual Health Questionnaire and 44% of their patient cohort noted subjective improvements in their erectile dysfunction following microsurgical varicocelectomy ^56^.

Thus, evidence seems to suggest a potential benefit to performing varicocelectomy in men with hypogonadism. In fact, a recent analysis found that hypogonadism diagnosis was a predictor of undergoing varicocele repair (OR 2.00, 95% CI 1.57 to 2.55), which may indicate increased acceptance of this indication for surgery ^57^. In summary, more studies are necessary to determine whether improvement following varicocelectomy is durable and would obviate the need for testosterone replacement in select individuals.

## Conclusions

As described above, a growing body of evidence seems to support varicocelectomy in cases of NOA, elevated DNA fragmentation, and hypogonadism. It is important to note a few limitations of these data. First, in the vast majority of the described studies, a microsurgical technique was used and in multiple large retrospective studies this technique has been shown to be safe and effective ^58– 60^. However, although it is perhaps intuitive to apply these results to other techniques, such as embolization, more studies are needed to establish the same benefits in regard to these select patient populations.

Second, most of the data summarized report benefits via surrogate outcomes, and a limited number of studies describe pregnancy rates and symptomatic improvements, for example. Alterations in surrogate outcomes may not translate to meaningful clinical outcomes.

Notwithstanding these limitations, by presenting available data this article may guide providers in sharing these data with patients in order to help them make an informed decision.
